# Scrub typhus, a salient threat: Needs attention

**DOI:** 10.1371/journal.pntd.0011427

**Published:** 2023-06-29

**Authors:** Kamran Zaman

**Affiliations:** Scientist E, Department of Microbiology and Molecular Biology, Indian Council of Medical Research - National Institute of Traditional Medicine Belagavi (ICMR-NITM Belagavi), Karnataka, India; All India Institute of Medical Sciences - Bathinda, INDIA

## Abstract

Scrub typhus (ST) infection is one of the most significant causes of acute undifferentiated febrile illness, and its prevalence has been increasing across the globe. Clinical suspicion and growing clinical understanding among healthcare professionals have resulted in the rapid diagnosis and effective management. Since ST has the potential to cause multiorgan failure and a higher mortality rate, it is critical to enhance surveillance, make rapid diagnosis, and administer antibiotics appropriately.

Scrub typhus (ST) is an acute fever sickness caused by the bacteria *Orientia tsutsugamushi* and spread by the larvae (chiggers) of *Leptotrombidium* mites. ST is endemic in the tsutsugamushi triangle, which stretches from southeastern Asia to the Pacific and has lately been recorded from various parts of the world outside the tsutsugamushi triangle [1]. Globally, it poses a threat to 1 billion people and affects 1 million people annually. Clinical symptoms of ST vary, ranging from acute undifferentiated febrile illness (AUFI) to multiorgan failure [1,2]. In Asia, seroepidemiological data showed seroprevalence ranging from 9.3% to 27.9% and a significant apparent increase in the incidence of the disease. The median fatality rate for untreated ST and treated ST varied as 6.0% versus 1.4%. High fatality rates are noted in patients with central nervous system involvement (13.6%) and multiorgan dysfunction (24.1%), and, also, high risk of miscarriage and poor neonatal outcomes have been noted in ST infections in pregnancy [1]. According to reports, ST has been the most prevalent reemerging rickettsia infection in India [2–4]. However, while most of the ST reporting comes from tertiary care facilities, teaching hospitals, and academic researchers in specified geographical areas, which means that they do not accurately reflect the disease’s prevalence, particularly in India’s rural areas, and there is still a dearth of comprehensive data on its prevalence in India [2–4].

Diverse risk factors for infection acquisition have been identified in various regions of the world, with few studies from India [5–8]. Comprehensive research is required to determine the risk factors for contracting an infection in the Indian context. ST is a neglected zoonotic disease spread by vectors, and its geographic range is growing. It has been investigated extensively how vectors and reservoirs contribute to the spread of the disease [9,10]. Entomological investigations showed that *O*. *tsutsugamushi* naturally infected small animal hosts and vector mites, according to research by Sadanandane C and colleagues. During the monsoon and post-monsoon seasons, the *Leptotrophombidium deliense* mite was widely distributed on *Suncus murinus* (shrew), and *O*. *tsutsugamushi* serotypes Gilliam, Karp, and TA678 were found to be in circulation through phylogenetic research [9–11]. Public health control measures for ST control are hindered by the complicated course of disease and poorly understood disease ecology.

Due to the varied range of clinical symptoms and the lack of clinical suspicion at the time of presentation, the diagnosis is often not established. Even though eschar is pathognomonic, ST cases seldom have it identified. The eschars are more common in adults compared to children where organomegaly, especially hepatomegaly, is often noted [1,2,4]. The currently used diagnostic techniques like serology, molecular, and culture require highly advanced infrastructures that are not always available in resource-constrained settings. Despite having a lesser sensitivity, the Weil–Felix test is still commonly used in small, outlying labs, and Immunoglobulin M enzyme-linked immunosorbent assay (ELISA) is considered a reliable diagnostic tool despite immunofluorescence assay (IFA) being the gold standard for ST diagnosis [2,12]. These diagnostic difficulties further contribute to not establishing a diagnosis of ST, which, in turn, contributes to the scant data available to estimate its burden. To broaden the diagnostic window toward early infection periods, IFA being substituted by the more objective ELISA combined with nucleic acid amplification assays is evolving [12]. There is no point-of-care tests that are comparable to IgM ELISA. Studies have shown that regional cutoffs could be included to make the commonly used ELISA more sensitive and specific [13,14].

Although ST poses a threat to over a billion people, the disease can be treated in an early stage with appropriate antibiotics. However, trials on ST treatments are few and often with small sample sizes. Chloramphenicol, azithromycin, rifampicin, and tetracycline all appear to be efficient [1,2,15]. Doxycycline and azithromycin have demonstrated good effectiveness in avoiding the progression of AUFI to acute encephalitis in children who come with acute fever illness in remote health facilities with high ST endemicity [16]. Furthermore, reports of ST infections in northern Thailand poorly responding to doxycycline has troubled health experts; vigilance on this aspect is much needed because of antibiotic misuse [17]. In India, a recent multicenter, double-blind, randomized, controlled trial by Varghese and colleagues demonstrated that intravenous doxycycline and azithromycin combination therapy was a more effective therapeutic choice than monotherapy with either drug alone for the management of severe ST [18].

Developmental delay, behavioral abnormalities, and neurological impairments are reported by nearly half of pediatric infective encephalitis survivors [19]. Acute disseminated encephalomyelitis, cerebellitis, transverse myelitis, cerebral venous sinus thrombosis, parkinsonism, cranial nerve palsies, opsoclonus-myoclonus syndrome, Guillain–Barré syndrome, and many other neurological syndromes have recently been described as being associated with ST. A study by Gangwar and colleagues showed 1/5th of the cases with significant disability post-ST infection showed impairment in the domains of cognition and behavior [20]. ST being reported as a leading agent in central nervous system infections, it’s important to understand in detail the neurologic manifestation, pathological damage, and its long-standing sequelae [21].

Sensitizing practitioners in the private sector regarding the timely initiation of appropriate antibiotics early in suspected cases of ST would also be necessary to prevent complications. Point-of-care tests or diagnostic assays at the primary healthcare level must also be implemented as part of a long-term plan to support early treatment at remote healthcare facilities. Conducive environmental factors make vector control measures challenging. According to research by Thangaraj and colleagues, people who practice open defecation, children who live, play, or visit in fields, people who keep firewood inside their homes, and people who handle livestock feed are more likely to contract ST in Indian scenario [5]. Therefore, providing community and household toilets through government flagship programs, as well as behavior change communication about avoiding open defecation, help to reduce such infections in the area. It is also crucial to screen the pediatric cohort of AUFI with seizures for ST, diagnose neurological deficits early, and initiate the appropriate rehabilitative therapy for encephalitis cases. To track the trend as well as the outcomes of various control measures, ST surveillance should continue in endemic regions [5,11].
